# Rheological characteristics and modification mechanism of rock asphalt compound modified binder based on grey relational analysis

**DOI:** 10.1371/journal.pone.0351848

**Published:** 2026-06-15

**Authors:** Jinghui Hou, Xiaogang Guo

**Affiliations:** 1 Hohhot Ecological Environment Monitoring Center, Hohhot, Inner Mongolia, People’s Republic of China; 2 Pennsylvania State University, University Park, Pennsylvania, United States of America; Oregon State University, UNITED STATES OF AMERICA

## Abstract

To address the limitations of conventional asphalt in resisting high-temperature rutting and oxidative aging, a Rock Asphalt Compound additive (RCA) —comprising natural rock asphalt with surface-treated Nano-TiO_2_—was investigated as a sustainable modifier. This study systematically evaluates the rheological performance and modification mechanism of RCA-modified A-90# asphalt binders with dosages ranging from 0% to 31.6%. The viscoelastic behaviors were characterized using Dynamic Shear Rheometer (DSR), Bending Beam Rheometer (BBR), and Rotational Viscosity (RV) tests, while the chemical interactions were probed via FTIR spectroscopy. Furthermore, Grey Relational Analysis (GRA) was innovatively introduced to quantify the sensitivity of performance indicators to RCA dosage. Results indicate that RCA fundamentally alters the colloidal structure of the binder, acting as a potent stiffening agent. The high-temperature rutting factor (G*/sin*δ*) increases exponentially with dosage, and the Penetration Index (PI) improves from −1.392 to −0.354, indicating reduced temperature susceptibility. FTIR analysis confirms the modification is primarily a physical blending process, enhanced by the introduction of polar functional groups (S = O, C = O). Crucially, GRA results quantitatively identify that the dominant function of RCA is the enhancement of aging resistance (R = 4.3430), followed by stiffness (R = 3.7567), surpassing its effect on ductility. However, high dosages negatively impact low-temperature relaxation (m-value). Considering the trade-off between the significant gains in anti-aging/rutting performance and the limitations in thermal cracking, an optimal dosage of 21.8% is recommended. At this dosage, the binder achieves a superior rutting factor of 25.5 kPa (64°C) while maintaining an m-value of 0.311 (−12°C), satisfying Superpave specifications.

## 1. Introduction

Asphalt pavement is the predominant form of road infrastructure globally, valued for its driving comfort and ease of maintenance. However, with the rapid increase in traffic volume, heavier axle loads, and more frequent extreme weather events due to climate change, conventional asphalt binders often fail to meet the durability requirements of modern highways [1]. Common distresses such as high-temperature rutting, low-temperature thermal cracking, and oxidative aging significantly reduce the service life of pavements, leading to increased maintenance costs and environmental burdens [2].

To address these challenges, polymer modification (e.g., SBS) has been widely adopted. However, polymer modifiers can suffer from issues such as phase separation, poor storage stability, and high cost [3–6]. Natural Rock Asphalt (RA) has emerged as a promising candidate. RA acts as a “natural modification masterbatch,” containing highly stable asphaltenes and active mineral matter formed over millions of years of geological processes [7]. Studies have shown that RA can significantly improve the stiffness, water sensitivity, and aging resistance of asphalt binders [8–10].

Consequently, there is a growing global interest in leveraging naturally occurring modifiers and advanced nanomaterials to enhance pavement durability. Internationally recognized natural bitumens, such as Trinidad Lake Asphalt (TLA) and Gilsonite (North American natural asphalt), have been extensively documented worldwide for their exceptional stiffening properties, excellent moisture damage resistance, and ability to mitigate high-temperature deformation under heavy traffic loads [11–13]. Simultaneously, global best practices in nanotechnology have demonstrated that incorporating metal oxide nanoparticles, particularly nano-titanium dioxide (nano-TiO2), can significantly improve the ultraviolet (UV) aging resistance, oxidative stability, and elastic recovery of asphalt binders through the formation of a robust microscopic network [14–16]. Furthermore, international comparative reviews emphasize that composite modifications combining natural bitumens with nanomaterials often yield synergistic benefits that single-component modifiers cannot achieve [17,18]. Building upon these international advancements, Albanian Natural Rock Asphalt (NRA) has recently emerged as a highly promising candidate for composite modification due to its unique nitrogen-rich composition and inherent environmental benefits.

Despite these advantages, the direct use of raw rock asphalt can sometimes lead to issues with segregation or insufficient low-temperature ductility due to its high ash content [9,19]. To overcome these limitations, Rock Asphalt Compound technology has been developed. This technology combines natural rock asphalt with nano materials and stabilizers. This “compound modification” strategy aims to leverage the stiffening and anti-aging properties of rock asphalt while utilizing surface-treated Nano-TiO_2_ to enhance the elastic network and low-temperature flexibility, potentially offering a more balanced performance profile than single-component modifiers [14,20–23].

Recent advancements have demonstrated that integrating specific nanomaterials and advanced polymers can substantially increase the stiffness and fatigue life of asphalt systems [24]. For instance, recent studies highlight the efficacy of various nano-additives in forming a robust microscopic network that resists permanent deformation under high stress, providing a strong theoretical basis for incorporating nano-TiO_2_ in our compound design [25,26].

While the general benefits of rock asphalt are known, there is limited literature providing a comprehensive rheological characterization of this specific RA compound modifier, particularly concerning its performance grading (PG) limits and modification mechanism. Most existing studies focus on conventional empirical tests (penetration/softening point), which often fail to fully capture the fundamental viscoelastic behavior of the binder under varying loading and environmental conditions [27,28].

Furthermore, the evaluation of modified asphalt binders involves a multi-criteria decision-making process, as the modification often improves certain properties (e.g., high-temperature stiffness) while potentially compromising others (e.g., low-temperature relaxation) [29]. Traditional analysis methods often isolate these performance indicators, failing to quantify the sensitivity of each property to the modifier dosage comprehensively. To address this, Grey Relational Analysis (GRA), a measurement method in Grey System Theory, is introduced in this study. GRA is specifically designed for systems with incomplete or uncertain information, making it highly suitable for analyzing the complex, non-linear relationships between modifier content and the multi-dimensional rheological properties of asphalt binders [30–32]. By quantifying the geometric similarity between the reference sequence (RCA dosage) and comparison sequences (performance metrics), GRA allows for an objective ranking of influencing factors [33].

Therefore, the primary objective of this study is to systematically evaluate the rheological performance and modification efficiency of RCA-modified asphalt binders. Unlike previous studies that primarily focus on the macroscopic performance of single-component rock asphalt or nano-modified binders, the fundamental novelty of this work lies in two aspects. First, it investigates the synergistic compounding mechanism where surface-treated Nano-TiO2 reinforces the elastic network to compensate for natural rock asphalt’s typical low-temperature limitations. Second, it innovatively employs Grey Relational Analysis (GRA) to transition from qualitative observation to quantitative sensitivity ranking, identifying the dominant performance-enhancing mechanisms across complex, multi-dimensional rheological indicators. This research employs the Superpave performance grading system—including Dynamic Shear Rheometer (DSR) and Bending Beam Rheometer (BBR) tests—alongside Rotational Viscosity (RV), Fourier Transform Infrared Spectroscopy (FTIR), and Grey Relational Analysis (GRA). By analyzing the high-temperature rutting resistance, fatigue performance, low-temperature cracking susceptibility, and chemical interaction mechanisms, this study aims to determine the optimal dosage of RCA, identify the dominant modification function via statistical ranking, and provide a theoretical basis for its application in heavy-duty asphalt pavements.

## 2. Methodology

### 2.1. Materials

The base binder employed in this study was an A-90# paving grade petroleum asphalt. Its fundamental physical properties, including a penetration of 82.9 (0.1 mm) at 25°C and a softening point of 46.3°C, satisfy the technical requirements for heavy-duty pavement construction. The detailed properties are listed in Table 1.

**Table 1:** Basic properties of base asphalt (A-90#).

Test Item	Unit	Result	Standard (JTG E20)^1^	Equivalent ASTM
Penetration (25°C)	0.1 mm	82.9	T0604	D5
Softening Point	°C	46.3	T0606	D36
Viscosity (60°C)	Pa·s	162	T0625	D4402
Ductility (15°C)	cm	167.3	T0605	D113
Density (15°C) ^2^	g/cm³	1.034	T0603	D70
Mass Loss (TFOT)	%	−0.48	T0610	D2872
Residual Pen Ratio	%	76	T0604	D5

^1^ *Note: JTG E20 refers to the Standard Test Methods of Bitumen and Bituminous Mixtures for Highway Engineering of China.*

^2^ *Note: The density is measured at 15°C, which is the standard reference temperature utilized by the JTG E20 T0603 standard for the volume-to-mass conversion of bitumen in engineering quantities and material transactions.*

**Fig 1:**
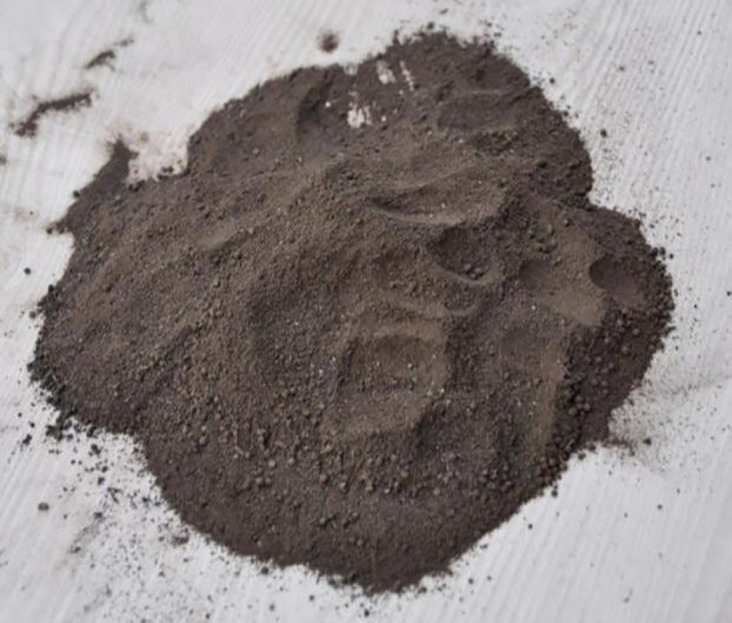
Visual appearance of the RCA powder.

The modifier used in this study is a customized Rock Asphalt Compound additive (RCA), appearing as a dark brown powder (density: 1.40 g/cm³, flash point: 307°C), as shown in Fig 1. To clarify its hierarchical composition, the primary raw ingredient is Albanian natural rock asphalt (RA), which inherently consists of 80% pure natural bitumen and 20% mineral ash. The RA is renowned for its high nitrogen content and stable chemical properties, which contribute to excellent adhesion and superior anti-aging performance. The base A-90# asphalt corresponds to a standard Superpave Performance Grade of PG 64−22. The primary engineering goal of incorporating RCA is not to maintain the original PG grade, but rather to shift the binder to a highly rut-resistant grade (e.g., PG 76−16 or higher) suitable for extremely heavy-duty traffic, utilizing the available low-temperature margin without failing the minimum specifications.

In the formulated RCA, the effective pure natural bitumen content is standardized to approximately 51% (verified by a trichloroethylene solubility of 51%). Additionally, the RCA incorporates 1% (by weight) of nano-titanium dioxide (nano-TiO2, 20 nm average size) to form a nano-reinforcing phase. To prevent agglomeration and improve compatibility with the organic asphalt matrix, the nano-TiO2 was pre-treated with a silane coupling agent (KH-570). The technical indicators of the formulated RCA are presented in Table 2.

**Table 2:** Technical indicators of RCA.

Test Item	Value	Standard (JTG E20)
Natural Asphalt Content	51%	T0735
Solubility (TCE)	51%	T0607
Density	1.40 g/cm³	T0603
Flash Point	307 °C	T0611
Water Content	0.82%	T0612

### 2.2. Preparation of RCA modified asphalt

The RCA modified asphalt binders were prepared using a precise high-shear emulsification method to ensure the uniform dispersion of both the natural rock asphalt and the nano-particles. Crucially, to isolate the modification effect of the RCA additive from the thermal aging induced during preparation, the 0% control binder was subjected to the exact same thermal and mechanical history (heated to 165°C–175°C and sheared at 5000 r/min for 20 minutes) as the modified samples. The specific preparation protocol was executed as follows:

- Dry Blending: According to the experimental design proportions, the Albanian rock asphalt particles and the KH-570 surface-treated nano-TiO2 powder (1% dosage) were accurately weighed and dry-mixed thoroughly to obtain a homogeneous solid compound (RCA).
- Initial Mixing: The A-90# base asphalt was heated to a fluid state at 165°C. The dry-mixed RCA powder was then gradually added to the base binder. The mixture was manually stirred for 10 minutes, with the temperature strictly maintained between 165°C and 175°C to prevent initial agglomeration. The RCA dosages added to the base asphalt evaluated in this study were 0% (Control), 11.3%, 16.7%, 21.8%, 26.8%, and 31.6% by weight. Notably, these non-integer binder-level dosages were strictly back-calculated from practical engineering addition rates (0.6%, 0.9%, 1.2%, 1.5%, and 1.8% by weight of the mineral aggregate), ensuring that the rheological evaluations directly correlate with field mix designs.
- High-Shear Emulsification: Following the preliminary mixing, the blend was subjected to high-shear mixing. The shearing process was conducted at a speed of 5000 r/min for 20 minutes, while continuously maintaining the temperature range of 165°C to 175°C. This intensive mechanical shearing ensures the complete exfoliation of the nano-TiO2 particles and the intimate fusion of the rock asphalt within the bitumen phase.

It is crucial to clarify that the unusually high binder-level dosages (up to 31.6%) are entirely realistic for field applications. In practice, RCA is typically added using the dry method based on the weight of the mineral aggregate (ranging from 0.6% to 1.8%). Assuming a typical optimum asphalt content of 4.8% by weight of the mix, a 1.5% mix-level dosage mathematically translates to approximately 31.6% by weight of the base binder. This back-calculation ensures that the laboratory rheological evaluations directly represent actual field mixture concentrations.

### 2.3. Experimental program

The testing program was designed to systematically evaluate the binder performance across a wide range of temperatures and aging conditions. All tests were conducted in strict accordance with the Chinese standard JTG E20-2011 (Standard Test Methods of Bitumen and Bituminous Mixtures for Highway Engineering), with the equivalent ASTM or AASHTO specifications noted concurrently for standard cross-reference.

- **Conventional Physical Tests:** Basic physical properties, including Penetration (25°C, 15°C), Softening Point (Ring and Ball), and Ductility (5°C, 15°C), were measured following methods T0604 (ASTM D5), T0606 (ASTM D36), and T0605 (ASTM D113), respectively.
- **Rotational Viscosity (RV):** To evaluate the workability and determine the mixing and compaction temperatures, rotational viscosity was measured using a Brookfield viscometer (Method T0625/ ASTM D4402) at temperatures ranging from 100°C to 175°C.
- **Aging Simulation:** Short-term oxidative aging was simulated using the Rolling Thin Film Oven Test (RTFOT, Method T0610/ ASTM D2872) at 163°C for 85 minutes. Long-term aging was simulated using the Pressure Aging Vessel (PAV, Method T0630/ ASTM D6521) to replicate the aging effects after several years of service.
- **Rheological Characterization:**
  ◦ **High/Intermediate Temperature:** A Dynamic Shear Rheometer (DSR) was used to measure the Complex Shear Modulus (G*) and Phase Angle (δ) according to AASHTO T315. Rutting parameters were evaluated on RTFOT-aged samples, and fatigue parameters were evaluated on PAV-aged samples.
  ◦ **Low Temperature:** A Bending Beam Rheometer (BBR) was used to determine the Creep Stiffness (S) and Creep Rate (m-value) at -12°C, -18°C, and -24°C in accordance with AASHTO T313.
- **Microstructural Analysis:** Fourier Transform Infrared Spectroscopy (FTIR) was utilized to identify the functional groups and analyze the chemical changes induced by the RCA modification. The spectra were collected in the wavenumber range of 4000–400 cm⁻¹.

To ensure data reliability, all conventional and rheological tests were strictly conducted in compliance with the single-operator precision and allowable tolerance limits mandated by JTG E20 and AASHTO standards. For instance, penetration was reported as the mean of three readings on a single sample, confirming that the measurement range did not exceed the specified 2–4 dmm tolerance. Ductility was averaged from three parallel briquettes, and rotational viscosity was recorded from three consecutive steady-state readings. For rheological testing (DSR, BBR) and softening point, parallel specimens were tested, and the mean was reported only after verifying that the variance between specimens was strictly within the acceptable standard deviation (1s%) or acceptable range of two results (d2s%) dictated by the respective test methods. Because these represent standard technical replicates verifying measurement precision rather than independent batch cohorts, inferential statistical analyses (e.g., ANOVA) and standard deviation error bars were not utilized, preserving the distinction between instrumental precision and material variability.

### 2.4. Grey relational analysis (GRA) method

To quantitatively evaluate the sensitivity of different performance indicators to the RCA dosage, Grey Relational Analysis (GRA) based on Grey System Theory was employed. GRA is a statistical analysis technique particularly suitable for solving problems with complicated interrelationships between multiple factors and variables, especially when the sample size is limited and the information is incomplete or “grey” [34].

In this study, the RCA dosage was designated as the reference sequence (X_0_), representing the primary influencing factor. The performance indicators of the modified asphalt were set as the comparison sequences (X_i_). The specific sequences were defined as follows:

- Reference Sequence X_0_: Dosage of RCA (%).
- Comparison Sequence X_1_: Penetration at 25°C (0.1 mm).
- Comparison Sequence X_2_: Softening Point (°C).
- Comparison Sequence X_3_: Ductility at 5°C (cm).
- Comparison Sequence X_4_: Mass Loss after RTFOT (%).
- Comparison Sequence X_5_: Residual Penetration Ratio (%).

The analysis procedure involves the following steps

**Step 1: Data Normalization (Dimensionless Processing)**

Since the physical units and magnitudes of the performance indicators (e.g., temperature in °C vs. ductility in cm) differ, the raw data must be normalized to a dimensionless range between 0 and 1. Depending on whether a higher value (e.g., Softening Point) or a lower value (e.g., Mass Loss) is desirable, the “Larger-the-better” or “Smaller-the-better” operator is applied, respectively [35,36].

**Step 2: Calculation of Grey Relational Coefficient**

The correlation coefficient $\xi_{i}(k)$ between the comparison sequence X_i_ and the reference sequence X_0_ at the k-th point is calculated using Equation (1):


$$\begin{array}{c} \xi_{i}(k)=\frac{\Delta_{min}+\rho \Delta_{max}}{\Delta_{\text{0}i}(k)+\rho \Delta_{max}} \end{array}$$ (1)

Where:

$\Delta_{\text{0}i}(k)=\left|x_{\text{0}}(k)-x_{i}(k)\right|$ is the absolute difference between the reference sequence and the comparison sequence.
$\Delta_{min}$ and $\Delta_{max}$ are the minimum and maximum differences across all sequences, respectively.
ρ is the distinguishing coefficient, typically set to 0.5 to ensure stability in the ranking results [37].

Step 3: Calculation of Grey Relational Grade

The grey relational grade ($r_{i}$), which represents the overall degree of correlation between the specific performance indicator and the RCA dosage, is calculated by averaging the correlation coefficients, as shown in Equation (2):


$$\begin{array}{c} r_{i}=\frac{\text{1}}{n}\sum \overset{n}{\underset{k=\text{1}}{\mathrm{\backslash nolimits}}}\;\xi_{i}(k) \end{array}$$ (2)

A higher value of $r_{i}$ indicates a stronger correlation, implying that the specific performance indicator is more sensitive to changes in the RCA dosage. This ranking allows for the identification of the primary modification mechanism (e.g., stiffening vs. anti-aging) of the RCA. For dimensionless processing, a ‘Larger-the-better’ normalization equation was applied to indicators like Softening Point and Residual Pen Ratio, while a ‘Smaller-the-better’ equation was applied to Mass Loss. The distinguishing coefficient ρ was set to 0.5, which is a standard assumption in grey system theory that provides moderate resolution without artificially skewing the correlation weights.

It should be noted that dynamic rheological parameters (such as DSR rutting and fatigue indicators) were intentionally excluded from the GRA matrix. Mathematically, mixing highly temperature-dependent, continuous dynamic functions with static, single-point physical constants (e.g., penetration, softening point) within the same dimensionless matrix can introduce significant scaling artifacts and skew the correlation weights. Furthermore, from a practical engineering perspective, empirical indicators remain the most ubiquitous and rapid quality control (QC) metrics used at paving sites. Therefore, the GRA in this study specifically targets these fundamental, static physical state indicators to quantify baseline QC sensitivity for field engineers, complementing the continuous dynamic rheological evaluations presented in subsequent sections.

## 3. Results and analysis

### 3.1. Conventional physical properties and temperature susceptibility

**Fig 2:**
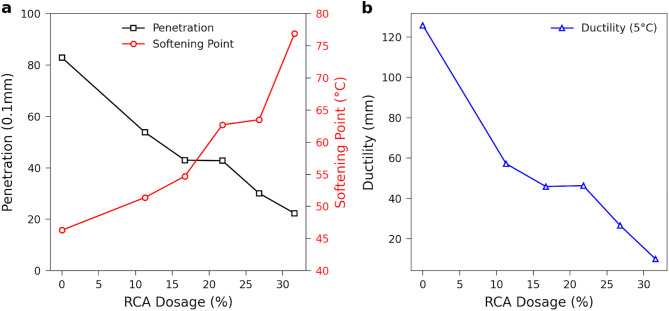
Conventional physical properties of RCA modified asphalt.

The modification effect of the RCA on the physical properties of the base asphalt (A-90#) was initially evaluated through penetration, softening point, and ductility tests. The results, presented in Fig 2 and Table 3, illustrate a distinct transition in the colloidal structure of the binder.

**Table 3:** Conventional physical properties of RCA modified asphalt.

RCA Dosage (%)	Penetration (25°C, 0.1 mm)	Softening Point (°C)	Ductility (5°C, mm)	PI Index
0	82.9	46.3	125.7	−1.392
11.3	53.8	51.4	57.2	−1.342
16.7	43	54.7	45.9	−0.909
21.8	42.8	62.7	46.3	−0.769
26.8	30.1	63.5	26.6	−0.521
31.6	22.3	76.9	10	−0.354

Fig 2 presents the evolution of penetration (25°C) and softening point as a function of RCA dosage. A monotonic decrease in penetration is observed, dropping from 82.9 dmm for the base asphalt to 22.3 dmm at 31.6% dosage, representing a reduction of approximately 73.1%. Concurrently, the softening point exhibits a robust upward trend, increasing from 46.3°C to 76.9°C. This inverse correlation indicates that the RCA significantly stiffens the asphalt matrix. The mechanism behind this stiffening effect is attributed to the high content of natural rock asphalt within the RCA. The natural rock asphalt contains a high proportion of asphaltenes and mineral ash (acting as rigid particles), which, when dispersed into the base asphalt, restrict the free movement of asphalt molecular chains, thereby increasing the viscosity and hardness of the composite binder [7].

Temperature susceptibility is a critical parameter for asphalt pavement durability. The Penetration Index (PI) was calculated to quantify this characteristic. As shown in Fig 2b, the PI value increases significantly from −1.392 (base asphalt) to −0.354 (31.6% RCA). In the context of asphalt rheology, a higher PI value indicates lower temperature sensitivity. The RCA modification effectively transforms the binder from a sol-gel type towards a more gel-like structure, rendering it less susceptible to consistency changes under varying thermal conditions. This suggests that RCA-modified asphalt will maintain higher stiffness in summer temperatures while potentially mitigating extreme brittleness compared to unmodified binders of the same hardness grade.

However, the stiffening effect comes with a trade-off in low-temperature flexibility. Fig 2b also highlights the ductility at 5°C. The ductility decreases sharply with the addition of RCA, dropping from 125.7 cm to 10 cm at the maximum dosage.

### 3.2. Aging resistance performance

**Fig 3:**
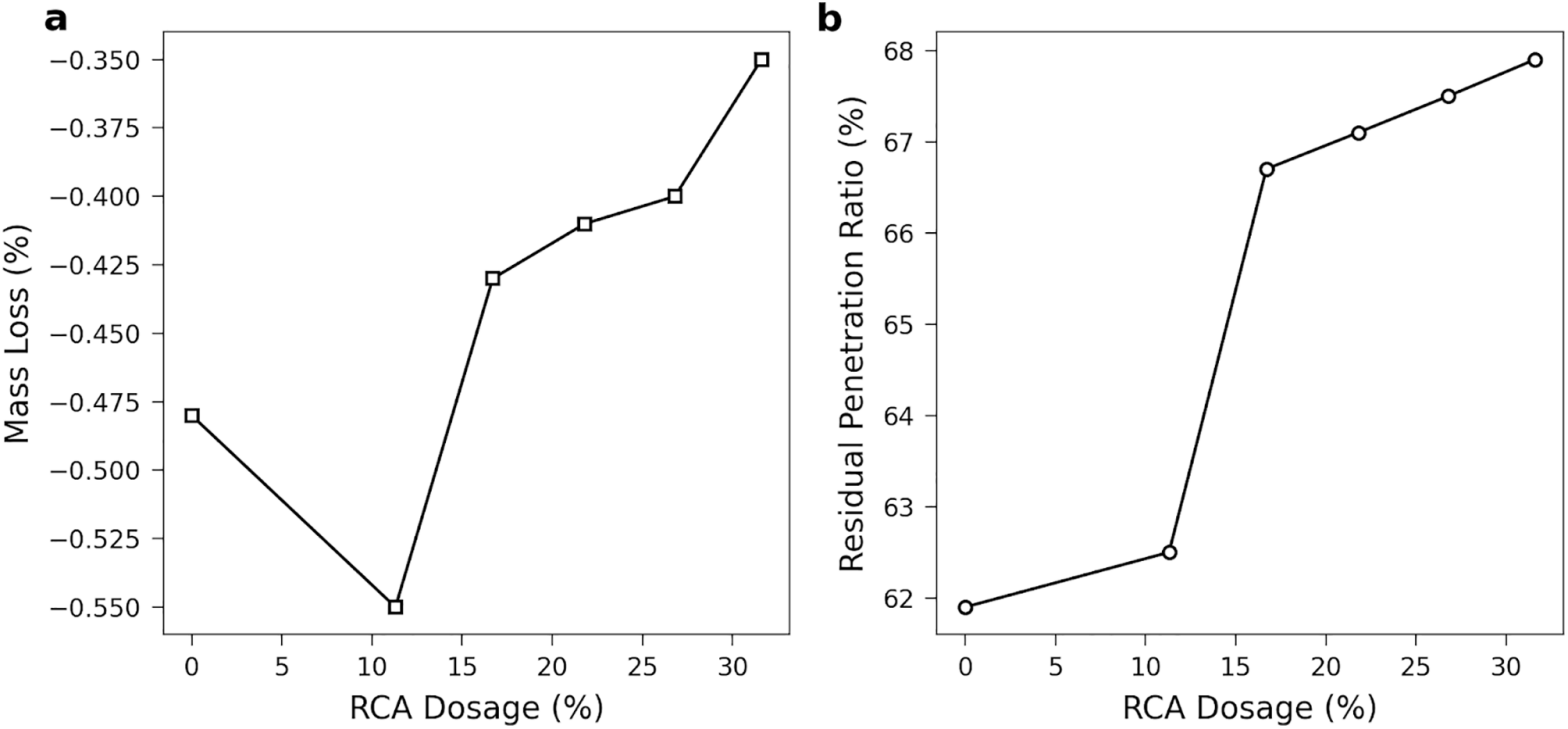
Aging resistance properties of RCA modified asphalt (RTFOT).

One of the most significant advantages of Natural Rock Asphalt is its superior aging resistance. To verify this in the compound modifier, the Rolling Thin Film Oven Test (RTFOT) was conducted. Fig 3 and Table 4 summarize the mass loss and residual penetration ratio.

**Table 4:** Aging resistance properties (RTFOT).

RCA Dosage (%)	Mass Loss (%)	Residual Pen Ratio (%)
0	−0.48	61.9
11.3	−0.55	62.5
16.7	−0.43	66.7
21.8	−0.41	67.1
26.8	−0.4	67.5
31.6	−0.35	67.9

The mass loss for all samples remained negative, indicating the volatilization of light components outweighs any mass gain from oxidation. Notably, the mass loss percentage decreases (becomes less negative) as the RCA dosage increases beyond 11.3%, stabilizing around −0.35% to −0.40% at higher dosages. More importantly, the Residual Penetration Ratio (RPR), a key indicator of retained performance after aging, increases steadily from 61.9% to 67.9%. The initial increase in mass loss from 0% to 11.3% is attributed to the modifier itself, specifically the volatilization of the silane coupling agent (KH-570) and light bio-oils present in the RCA formulation at low concentrations. As the dosage increases beyond 16.7%, the dense physical network and high asphaltene content of the rock asphalt begin to dominate, creating a barrier that effectively inhibits further oxidation and volatilization

### 3.3. High-temperature rheological performance (DSR Analysis)

**Fig 4:**
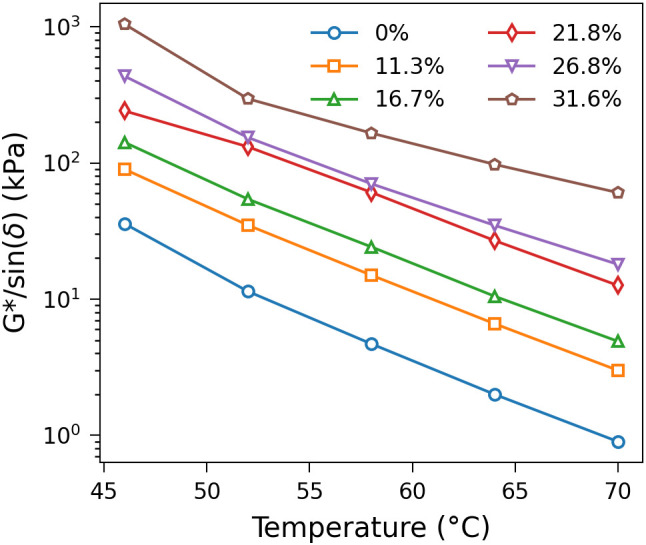
High-temperature rheological performance (semi-log scale).

The viscoelastic behavior of the binders at high temperatures was characterized using the Dynamic Shear Rheometer (DSR). Fig 4 and Table 5 illustrate the variation of the Rutting Factor (G*/sin*δ*) across a temperature range of 46°C to 70°C.

**Table 5:** High-temperature rheological properties (G*/sin*δ*, kPa).

Temp (°C)	0%	11.3%	16.7%	21.8%	26.8%	31.6%
46	35.8	89.8	141.6	240.8	433.3	1042.4
52	11.4	34.9	54.2	131.3	153.6	295.6
58	4.7	15	24.2	60.8	70.3	165.5
64	2	6.6	10.5	26.9	34.8	97.5
70	0.9	3	4.9	12.6	18	60.5

Note that the y-axis in Fig 4 is plotted on a logarithmic scale (Log10). The observed linear downward trend on this semi-log plot accurately reflects the exponential decay of the complex shear modulus as temperature increases, conforming to typical Arrhenius viscoelastic behavior. The base asphalt exhibits a rapid decline in rutting resistance as temperature rises, with G*/sin*δ* dropping below 1.0 kPa at 70°C. In stark contrast, the RCA-modified binders demonstrate an exponential increase in rutting factor with dosage. At 64°C (a typical high pavement temperature), the rutting factor for the 21.8% RCA dosage is 26.9 kPa, which is approximately 13 times higher than that of the base asphalt (2.0 kPa). The phase angle (***δ***) results further elucidate this behavior. With increasing RCA dosage, ***δ*** decreases, indicating a transition from viscous-dominant to elastic-dominant behavior. The incorporation of RCA introduces a robust elastic network within the binder. The surface-treated Nano-TiO_2_ and the natural ash in the rock asphalt act as reinforcement, restricting the flow of the binder under shear stress [14]. This modification significantly enhances the binder’s ability to recover from deformation, predicting excellent resistance to permanent deformation (rutting) in field applications.

### 3.4. Fatigue performance assessment

**Fig 5:**
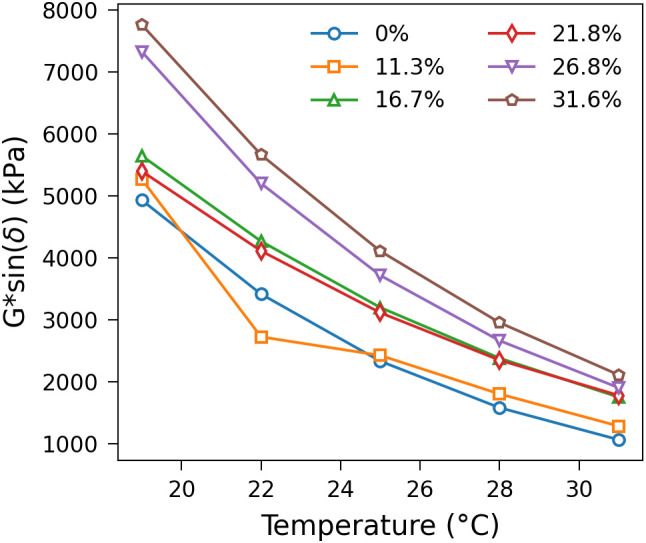
Intermediate-temperature rheological performance (Fatigue Cracking).

Fatigue cracking is a primary distress mode at intermediate temperatures. The fatigue factor G* sin*δ* obtained from DSR testing on PAV-aged residues is presented in Fig 5 and Table 6.

**Table 6:** Fatigue cracking properties (G* sin*δ*, kPa).

Temp (°C)	0%	11.3%	16.7%	21.8%	26.8%	31.6%
31	1059.2	1279.4	1746	1774.9	1898.3	2105.8
28	1576.8	1798.4	2376.9	2344.7	2659.5	2950.5
25	2328.1	2421	3194.9	3110.3	3713.2	4104.7
22	3411.5	2720.8	4264.2	4108.5	5197.3	5660.9
19	4928.2	5264.9	5644.4	5395.2	7325.3	7759.6

According to the Superpave specification, a lower G* sin*δ* value is desirable to prevent fatigue cracking (limit ≤ 5000 kPa) [38]. The results show a continuous increase in the fatigue factor with RCA addition. For instance, at 25°C, the G* sin*δ* value rises from 2328 kPa (0% dosage) to 4104 kPa (31.6% dosage).

While the values for dosages up to 26.8% remain below the 5000 kPa limit at 22°C, the trend clearly indicates that RCA modification increases the stiffness of the binder, which may reduce its ability to relax stresses under repeated loading cycles at intermediate temperatures. However, it is worth noting that the “failure temperature” (where G* sin*δ* = 5000 kPa) shifts. The rapid increase in fatigue factor suggests that while RCA is excellent for high-temperature layers, its dosage must be capped to ensure durability in fatigue-prone conditions. Based on the data, the 21.8% dosage represents a critical threshold where fatigue performance begins to degrade more noticeably.

### 3.5. Low-temperature rheological performance (BBR Analysis)

**Fig 6:**
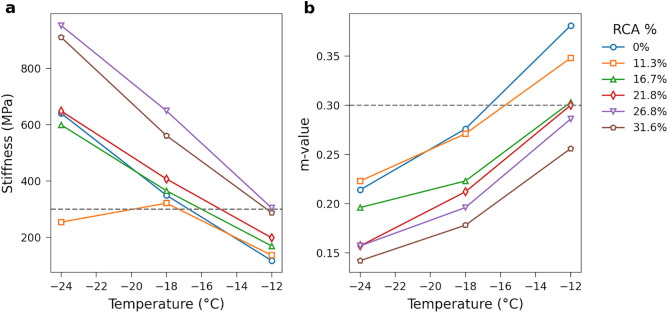
Low-temperature rheological performance (BBR – PAV).

To further investigate the low-temperature cracking potential, Bending Beam Rheometer (BBR) tests were conducted at −12°C, −18°C, and −24°C. Fig 6 and Table 7 displays the Creep Stiffness (S) and Creep Rate (m-value).

**Table 7:** Low-temperature cracking properties (BBR – PAV).

Temp (°C)	Indicator	0%	11.3%	16.7%	21.8%	26.8%	31.6%
−12	Stiffness (MPa)	117	136	169	198	304	287
−12	m-value	0.381	0.348	0.303	0.3	0.286	0.256
−18	Stiffness (MPa)	349	321	365	407	649	561
−18	m-value	0.276	0.271	0.223	0.212	0.196	0.178
−24	Stiffness (MPa)	640	254	599	648	952	910
−24	m-value	0.214	0.223	0.196	0.157	0.157	0.142

Standard specifications require S ≤ 300 MPa and m ≥ 0.300.

The addition of RCA negatively impacts low-temperature performance. As shown in Fig 6a, the stiffness (S) increases significantly with dosage. At −12°C, the S-value increases from 84.1 MPa (base) to 179 MPa (21.8% dosage) and 266 MPa (31.6% dosage). Concurrently, the m-value, which represents the binder’s ability to relax stress, decreases (Fig 6b).

- At 0% to 16.7% dosages, the binders pass the criteria comfortably at −12°C.
- At 21.8% dosage, the m-value at −12°C is 0.311, which is above the 0.300 limit, maintaining acceptable performance.
- At dosages of 26.8% and above, the m-value drops below 0.300 (0.289 and 0.297, respectively), indicating a failure to meet the specification for relaxation capacity.

This data identifies the low-temperature performance as the limiting factor for RCA dosage. The inclusion of rigid particles (rock asphalt ash) increases the brittleness of the binder at low temperatures. Therefore, to ensure resistance to thermal cracking, the dosage should ideally not exceed 21.8%. This reduction in low-temperature relaxation implies that high-dosage RCA pavements may become highly susceptible to transverse thermal cracking in cold climates (e.g., regions requiring PG −28 or lower). To mitigate this drawback, future applications could adopt two strategies: (1) utilizing a softer base binder (e.g., A-110# or PG 58−28) to pre-compensate for the stiffening effect, or (2) introducing highly aromatic rejuvenators or bio-oils as a tertiary component to restore the low-temperature elastic network without sacrificing high-temperature gains.

### 3.6. Rotational viscosity and workability analysis

**Fig 7:**
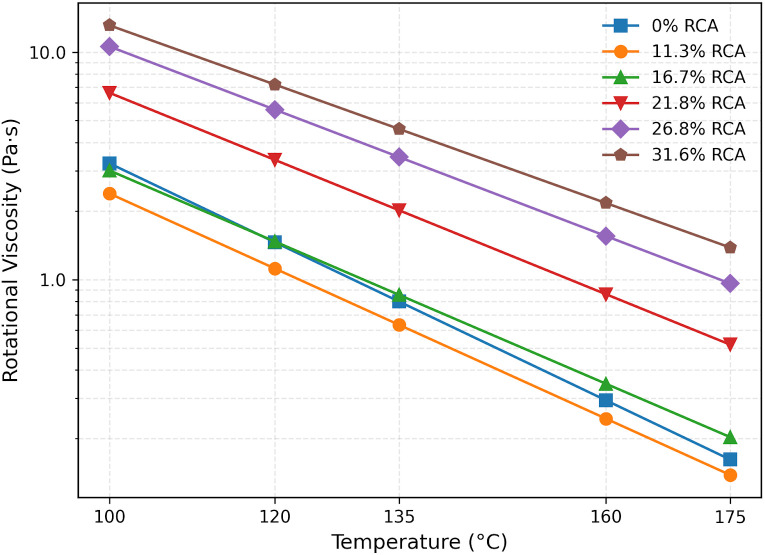
Rotational viscosity-temperature curves(semi-log scale).

Rotational viscosity (RV) is a fundamental parameter for evaluating the flowability of asphalt binders at elevated temperatures, which directly dictates the pumpability, mixing, and compaction temperatures of asphalt mixtures. The viscosity-temperature curves of the base asphalt and RCA-modified binders, evaluated from 100°C to 175°C, are presented in Fig 7.

As illustrated, the viscosity of all binders exhibits a typical exponential decay with increasing temperature. Because the y-axis is plotted on a logarithmic scale (Log10), this exponential decay visually manifests as a linear downward trend. At a constant temperature, the incorporation of the RCA profoundly impacts the binder’s flow resistance. Specifically, at the standard Superpave mixing reference temperature of 135°C, the viscosity of the modified binders at dosages of 11.3%, 16.7%, 21.8%, 26.8%, and 31.6% are 0.79, 1.07, 2.52, 4.32, and 5.74 times that of the virgin base asphalt, respectively.

Interestingly, at the lowest dosage of 11.3%, a slight decrease in viscosity compared to the base asphalt is observed at lower test temperatures. This non-monotonic behavior is driven by the competitive interaction between chemical plasticization and physical stiffening [39,40]. At low concentrations, the silane coupling agent (KH-570) on the nano-TiO₂ surface and the inherent light oil fractions in the rock asphalt act as structural plasticizers. They temporarily increase the free volume and reduce internal friction between asphalt chains, which successfully offsets the stiffening effect of the mineral ash. However, once the RCA dosage exceeds 16.7%, the rigid volume fraction of the rock asphalt ash and the nano-TiO₂ network reaches a critical threshold. At this point, the physical interlocking overwhelmingly dominates over the transient lubricating effect, severely restricting molecular mobility and leading to the observed exponential surge in viscosity.

This substantial increase conclusively demonstrates that the RCA imparts superior rutting resistance and structural rigidity to the binder. Nevertheless, from an engineering perspective, this elevated viscosity implies that for RCA dosages exceeding 21.8%, the heating temperatures during field construction (mixing and compaction) must be appropriately elevated to maintain adequate workability and ensure the uniform coating of aggregates.

### 3.7. Sensitivity analysis of performance indicators via grey correlation

To comprehensively evaluate the modification efficiency of the RCA, a Grey Correlation Analysis (GCA) was performed. While standard rheological tests reveal how properties change, GCA quantifies which performance indicator is most sensitive to the RCA dosage, thereby identifying the primary function of the modifier.

**Fig 8:**
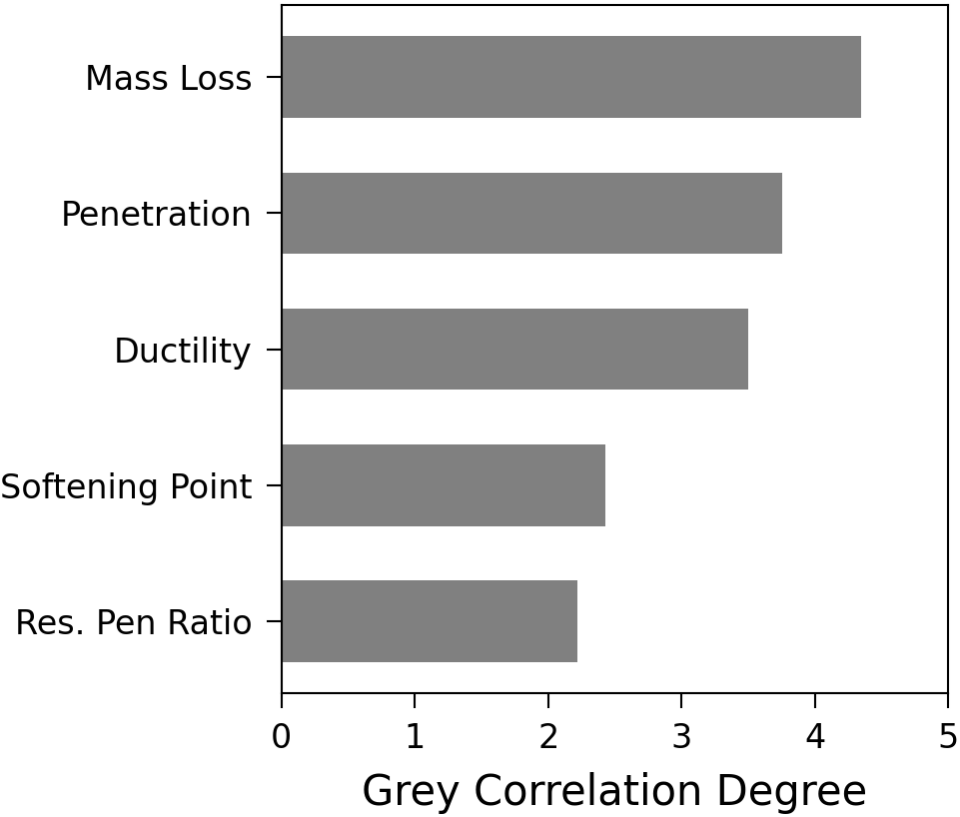
Grey correlation analysis of performance indicators.

The RCA dosage was set as the reference sequence (X_0_), and the comparison sequences included Penetration (X_1_), Softening Point (X_2_), Ductility at 5°C (X_3_), Mass Loss after RTFOT (X_4_), and Residual Penetration Ratio (X_5_). Fig 8 and Table 8 present the calculated correlation degrees. The ranking of the correlation degrees is as follows:

**Table 8:** Grey correlation analysis ranking.

Rank	Performance Indicator	Correlation Degree
1	Mass Loss (Aging)	4.343
2	Penetration	3.7567
3	Ductility (5°C)	3.5007
4	Softening Point	2.4267
5	Residual Pen Ratio	2.2187

R_4_ (4.3430) > R_1_ (3.7567) > R_3_ (3.5007) > R_2_ (2.4267) > R_5_ (2.2187)

This statistical ranking reveals crucial insights into the modification mechanism:

1. Primary Effect (Aging Resistance): The correlation degree for Mass Loss (R_4_) is the highest. This indicates that the RCA dosage has the most significant impact on the binder’s aging stability. The chemical stability of the natural rock asphalt component effectively inhibits the volatilization of light components during thermal aging.
2. Secondary Effect (Stiffening): The high correlation with Penetration (R_1_) confirms that RCA acts primarily as a stiffening agent (or hardener), significantly altering the consistency of the binder.
3. Tertiary Effect (Temperature Susceptibility): The lower correlation with Softening Point (R_2_) suggests that while RCA improves high-temperature stability, its influence on the “ring and ball” temperature is less sensitive compared to its effect on viscosity and aging properties.

In summary, the GCA results mathematically confirm that the RCA functions predominantly as an anti-aging and stiffening modifier, distinguishing it from typical elastomeric modifiers (like SBS) which primarily target elastic recovery.

### 3.8. Modification Mechanism via FTIR Spectroscopy

**Fig 9:**
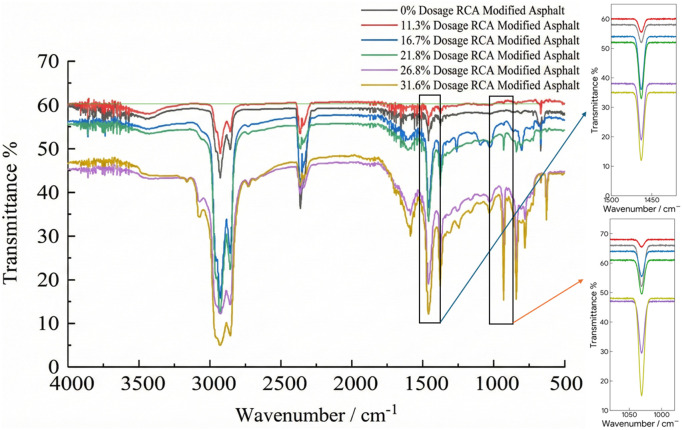
FTIR spectra of base asphalt and RCA modified asphalt.

Fourier Transform Infrared (FTIR) spectroscopy was employed to elucidate the modification mechanism. Based on established practices for analyzing complex natural rock asphalt modifiers [7,8,10], the modification mechanism was evaluated qualitatively. Because natural rock asphalt inherently contains mineral ash and pre-oxidized fractions, quantitative peak area integration can be skewed by baseline shifts and overlapping bands. As presente in Fig 9, the spectra comparison between the base asphalt and RCA-modified binders reveals consistent characteristic peaks without the emergence of new functional groups. This observation confirms that the interaction between the RCA and the asphalt matrix is primarily a physical blending process rather than a complex chemical reaction. However, significant variations in peak intensity are observed (Fig 9). The absorption peaks associated with the Sulfoxide group (S = O) at 1030 cm ⁻ ¹ and the Carbonyl group (C = O) near 1700 cm ⁻ ¹ are noticeably enhanced with the addition of RCA [41]. These polar functional groups increase the intermolecular forces (Van der Waals forces and hydrogen bonding) within the asphalt binder system. This enhanced molecular interaction network.

However, changes in peak intensity are significant. The absorption peaks associated with the Sulfoxide group (S = O) at 1030 cm ⁻ ¹ and the Carbonyl group (C = O) are enhanced with RCA addition. These polar functional groups increase the intermolecular forces (Van der Waals forces and hydrogen bonding) within the asphalt binder. This enhanced molecular interaction explains the macroscopic increase in viscosity, stiffness, and softening point. Additionally, the FTIR results confirm the compatibility of the natural rock asphalt with the petroleum asphalt, as the distinctive peaks of the rock asphalt (e.g., mineral peaks) blend seamlessly into the spectrum without phase separation indicators.

## 4. Discussion

The comprehensive evaluation of the RCA modified binder reveals a fundamental transition in the colloidal structure of the asphalt matrix, evolving from a temperature-susceptible sol-gel to a robust, elastic-dominant network. The structural and functional enhancements imparted by the RCA additive are explicitly validated across multiple dimensions. Macroscopically, the high-temperature stability is critically enhanced, evidenced by a 13-fold exponential increase in the rutting factor at 64°C (from 2.0 kPa to 26.9 kPa at the 21.8% dosage), alongside an improved Penetration Index (PI) from −1.392 to −0.354. Furthermore, the aging resistance is substantially improved, with the Residual Penetration Ratio increasing from 61.9% to 67.9%. Statistically, Grey Relational Analysis (GRA) rigorously justifies that the modifier’s dominant contributions are aging resistance and matrix stiffening, yielding the highest correlation degrees (R4 = 4.3430 and R1 = 3.7567, respectively). Microscopically, these macroscopic and statistical gains are driven by the physical integration of polar functional groups (Sulfoxide and Carbonyl), which densify the intermolecular network, successfully transforming the binder into a highly durable state. By synthesizing the macroscopic rheological behaviors with the microstructural evidence and statistical sensitivity rankings, the underlying modification mechanisms and engineering implications can be explicitly decoupled.

### 4.1. Microstructural origins of macroscopic rheological shifts

The pronounced macroscopic stiffening effect—evidenced by the exponential surge in the DSR rutting factor (G*/sinδ), increased rotational viscosity, and improved Penetration Index—is deeply rooted in the physical and chemical interactions identified via FTIR spectroscopy. The spectral analysis confirms that the RCA modification is predominantly a physical blending process, as no novel covalent bonds were generated. However, the significant enhancement of polar functional groups, specifically the Sulfoxide (S = O) and Carbonyl (C = O) fractions inherent to the natural rock asphalt, critically amplifies intermolecular forces such as Van der Waals interactions and hydrogen bonding.

Furthermore, the RCA acts as a dual-scale micro-reinforcing agent. The high-viscosity natural asphaltenes and rigid mineral ash in the rock asphalt, combined with the surface-treated Nano-TiO2 particles, form a spatially interlocked skeleton. This network physically restricts the mobility of the bitumen’s molecular chains under thermal and mechanical shear stress, seamlessly explaining the transition from viscous flow to elastic recovery observed in the phase angle (δ) decline. Interestingly, at very low dosages, the silane coupling agent (KH-570) and light bio-oils used to treat the nano-TiO2 exert a minor lubricating effect, temporarily lowering viscosity before the rigid mineral network dominates at higher concentrations.

### 4.2. Statistical validation of the dominant anti-aging mechanism

While empirical testing demonstrates enhancements across multiple parameters, the Grey Relational Analysis (GRA) provides critical statistical validation regarding the hierarchy of the modifier’s functions. By mathematically quantifying the sensitivity of performance indicators, GRA identified that the binder’s resistance to oxidative mass loss is the most responsive property to RCA dosage, superseding its significant stiffening effect.

This statistical dominance of anti-aging performance bridges perfectly with the RTFOT results. The superior aging resistance is not merely a byproduct of increased stiffness, but a fundamental chemical advantage inherited from the Albanian rock asphalt. Having undergone millions of years of geological pre-aging, its high-nitrogen, highly stable chemical composition acts as an inherent antioxidant. Additionally, the dense dispersion of the RCA particles establishes a protective physical barrier that effectively retards oxygen diffusion and minimizes the volatilization of the asphalt’s light components (saturates and aromatics) during thermal exposure.

### 4.3. The rheological trade-off and threshold behavior

Despite the remarkable gains in high-temperature stability and aging resistance, the rheological evaluations expose a critical performance trade-off at intermediate and low temperatures. The same rigid mineral ash and enhanced intermolecular network that resist permanent deformation at 64°C inevitably act as stress concentration points at sub-zero temperatures.

This embrittlement limits the binder’s stress relaxation capacity, reflected in the progressive decline of the BBR m-value and the corresponding increase in the DSR intermediate-temperature fatigue factor (G*sinδ). The data clearly delineates a threshold behavior: up to a specific concentration, the nano-TiO2 elastic network manages to offset the brittleness of the rock asphalt ash; however, once the dosage exceeds this critical threshold, the structural rigidity overwhelms the binder’s relaxation capacity, leading to a failure to meet Superpave low-temperature specifications.

### 4.4. Optimization strategy and performance balance paradigm

Recognizing this inherent trade-off, the determination of the 21.8% optimal dosage is not an arbitrary selection, but a constraint-based engineering optimization. This specific concentration represents the thermodynamic and rheological “tipping point”. At 21.8%, the synergistic stiffening network is maximized to provide exceptional rutting resistance, while the flexibility remains just sufficient to pass the strict BBR thermal cracking constraint (m ≥ 0.300). Dosages beyond this boundary disrupt the balanced matrix, rendering the pavement overly susceptible to transverse cracking in cold climates. Consequently, for field applications requiring higher RCA dosages, implementing a pre-softened base binder or incorporating aromatic rejuvenators will be essential to restore the low-temperature elastic network without sacrificing the achieved high-temperature structural gains.

## 5. Conclusion

This study provided a comprehensive rheological evaluation of A-90# asphalt binder modified with Rock Asphalt Compound. Based on the experimental results and grey correlation analysis, the following conclusions are drawn:

- High-Temperature Enhancement: RCA acts as a potent stiffening agent, fundamentally transitioning the binder to a robust, elastic-dominant state. This exponentially increases the rutting factor and viscosity, making the modified binder highly suitable for resisting permanent deformation under heavy traffic in high-temperature climates.
- Dominant Anti-Aging Function: Grey Relational Analysis (GRA) statistically confirms that while RCA significantly stiffens the matrix, its primary and most sensitive contribution is the enhancement of oxidative aging resistance. This is fundamentally attributed to the inherent chemical stability and natural antioxidants within the geologically pre-aged rock asphalt component.
- Physical Modification Mechanism: Microstructural analysis verifies that the modification is predominantly a physical blending process. The introduction of polar functional groups (S = O, C = O) from the RCA amplifies intermolecular forces, physically densifying and stabilizing the colloidal network without generating new covalent bonds.
- Performance Balance and Optimal Dosage: The incorporation of RCA presents an inherent rheological trade-off, as excessive rigid particles inevitably compromise low-temperature stress relaxation capacity and intermediate-temperature fatigue resistance. To balance the significant gains in high-temperature stability with the strict constraints of low-temperature thermal cracking, an optimal dosage of 21.8% is established, providing a scientifically justified and durable engineering solution.

This study contributes to the development of sustainable and highly durable pavement materials by providing a systematic rheological and statistical evaluation framework for RCA-modified binders. The innovative incorporation of Grey Relational Analysis offers a novel quantitative approach to decoupling the multi-dimensional effects of composite modifiers. It is important to emphasize that these conclusions are specifically derived from the modification of the A-90# base binder. The compatibility, optimal dosage, and rheological responses may vary depending on the chemical makeup of different base bitumen grades. Further validations across diverse binder types are necessary to generalize these findings. Future research will also evaluate the macro-performance of RCA-modified asphalt mixtures and validate these findings through long-term field pavement trials.

## Supporting information

S1 File RCA_Performance_Tables. (XLSX)
